# Serologic Status for Pandemic (H1N1) 2009 Virus, Taiwan

**DOI:** 10.3201/eid1701.100014

**Published:** 2011-01

**Authors:** Daniel Tsung-Ning Huang, Pei-Lan Shao, Kuo-Chin Huang, Chun-Yi Lu, Jen-Ren Wang, Shin-Ru Shih, Hsin Chi, Mei-Ru Lai, Chin-Yun Lee, Luan-Yin Chang, Li-Min Huang

**Affiliations:** Author affiliations: Mackay Memorial Hospital, Taipei, Taiwan (D.T.-N. Huang, H. Chi);; National Taiwan University Hospital, Taipei (P.-L. Shao, K.-C. Huang, C.-Y. Lu, M.-R. Lai, C.-Y. Lee, L.-Y. Chang, L.-M. Huang);; National Cheng Kung University, Tainan, Taiwan (J.-R. Wang);; Chang-Gung Memorial Hospital, Taipei (S.-R. Shih);; National Taiwan University, Taipei (L.-M. Huang)

**Keywords:** Pandemic (H1N1) 2009, viruses, influenza, neutralization assay, hemagglutination inhibition assay, serologic assays, vaccine, Taiwan, dispatch

## Abstract

We studied preexisting immunity to pandemic (H1N1) 2009 virus in persons in Taiwan. A total of 18 (36%) of 50 elderly adults in Taiwan born before 1935 had protective antibodies against currently circulating pandemic (H1N1) 2009 virus. Seasonal influenza vaccines induced antibodies that did not protect against pandemic (H1N1) 2009 virus.

As experts were predicting and warning of a new influenza pandemic (*1*), an influenza epidemic occurred in April 2009 in the United States and Mexico and resulted in a pandemic 2 months later. The etiologic agent was identified as pandemic (H1N1) 2009 virus. Worldwide, most patients infected with this virus were <25 years of age, and one third of serious cases were in persons <50 years of age (*2**,**3*).

The hemagglutinin gene of pandemic (H1N1) 2009 virus was shown to be derived from the 1918 swine influenza virus and contained other genes from human, avian, and swine influenza viruses from Eurasia (*2*). In this study, we evaluated levels of preexisting cross-reactive antibodies against pandemic (H1N1) 2009 virus produced after previous infection in children and adults in Taiwan. We also examined serologic changes after vaccination with seasonal nonadjuvanted influenza vaccine.

## The Study

Serum samples were obtained during a nationwide influenza vaccine serologic study in Taiwan that started in 2006. Children (<5 years of age), adults (20–49 years of age), older adults (50–74 years of age), and elderly adults (>75 years of age) were recruited. Serum samples were obtained immediately before and 3 weeks after intramuscular injection with 1 dose of nonadjuvanted, trivalent, inactivated influenza vaccine formulated for the 2008–09 Northern Hemisphere winter season (samples were obtained from some participants >75 years of age before and after receiving 1 dose of the vaccine formulated for the 2007–08 winter season).

Microneutralization (MN) and hemagglutination inhibition (HI) assays were performed according to the World Health Organization Manual on Animal Influenza Diagnosis and Surveillance (*4*). Using these assays with 0.75% guinea pig erythrocytes, we assayed samples for antibodies against A/California/07/2009 (H1N1) virus. Only prevaccination HI assays were conducted for children.

The seroprotection rate was defined as the percentage of serum titers >40 by HI or titers >160 by MN. The seroconversion rate was defined as the percentage of vaccine recipients whose serum HI titers or MN titers increased by at least 4-fold after vaccination. A p value <0.05 was considered significant. Stata software version 8.2 (StataCorp LP, College Station, TX, USA) was used for analysis.

A total of 176 participants (40 children, 36 adults, 50 older adults, and 50 elderly adults) were enrolled (Table). Few or no preexisting cross-reactive antibodies against pandemic (H1N1) 2009 virus were detected by HI assay in samples from children (prevaccination seroprotection rate 0%). As age increased, prevaccination seroprotection rates became higher for HI and MN assays. After vaccination, seroprotection rates and geometric mean titers measured by HI assay were essentially unchanged but increased significantly in the 3 adult groups when measured by MN assay (p<0.05). Seroconversion rates among all participants were low. Analyses of relationships between age and antibody titers are shown in the Figure.

**Table:** Geometric mean titers of antibodies and rates of seroprotection against pandemic (H1N1) 2009 virus before and after seasonal influenza vaccination, by age, Taiwan, 2007–2008*

Group, age, y	Prevaccination GMT (95% CI)	Prevaccination seroprotection rate, %	Postvaccination GMT (95% CI)	Postvaccination seroprotection rate, %	p value
Children <5, n = 40)					
HI	10.4 (9.9–10.9)	0	ND	ND	ND
Adults 20–49, n = 36					
HI	12.1 (10.7–13.7)	2.8	12.1 (10.7–13.7)	2.80	NS
MN	26.3 (20.9–32.8)	0	31.7 (25.0–40.3)	0	<0.05
Older adults 50–74, n = 50					
HI	16.7 (14.2–19.7)	16	16.7 (14.2–19.7)	16	NS
MN	59.0 (47.7–72.8)	20	74.6 (60.0–92.9)	32	<0.05
Elderly adults >75, n = 50					
HI	22.7 (19.5–26.4)	36	23.3 (19.8–27.4)	38	0.159
MN	85.7 (70.8–103.9)	32	107 (89.2–128.5)	44	<0.05
*GMT, geometric mean titer; CI, confidence interval; HI, hemagglutination inhibition; ND, not done; NS, not significant. MN, microneutralization. Mean ± SD ages for the 4 groups were 20.0 ± 11.3 mo for children, 34.5 ± 7.5 y for adults, 65 ± 6 y for older adults, and 79 ± 3.3 y for elderly adults.					

**Figure:**
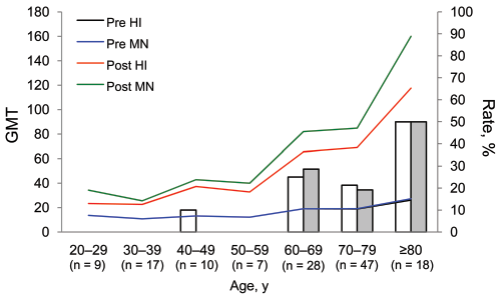
Seroprotection rates determined by hemagglutination inhibition (HI) assay (white bars) or microneutralization (MN) assay (gray bars) and geometric mean titer (GMT) of antibodies against pandemic (H1N1) 2009 virus in each 10-year age cohort, Taiwan, 2007–2008.

We log-transformed MN and HI titers, and used multiple regression, controlling for age groups to analyze the correlation between age and titer. Doubling of HI titers corresponded to an estimated 75% (p<0.01) increment in MN titers adjusted by age. When adjusted for HI titers, MN titers in older adults and elderly adults were 1.74× (p<0.01) and 2× (p<0.01), respectively, those in adults. Older adults and elderly adults with the same HI titers were more likely to have higher MN titers than adults (p<0.05, by ordinal logistic regression analysis).

## Conclusions

We found that children in Taiwan had few or no cross-reactive antibodies against pandemic (H1N1) 2009 virus. However, adults had some preexisting immunity to this virus. A major finding was that 18 (36%) of 50 elderly adults in Taiwan born before 1935 had protective antibodies against currently circulating pandemic (H1N1) 2009 virus. The seroprotection rate may be 50% in persons >80 years of age.

The MN assay showed that seasonal influenza vaccines generated large increases in geometric mean titers in vaccinees in all age groups. We suggest that seasonal influenza vaccines are likely to elicit a certain degree of cross-reactive antibodies against pandemic (H1N1) 2009 virus and may provide some level of protection. In persons who had no preexisting seroprotective titers against pandemic (H1N1) 2009 virus, the cross-reactivity produced was not sufficient to prevent disease; however, it may protect against the severe forms of the disease.

Hancock et al. (*5*) reported that only 4% of persons in the United State born after 1980 had preexisting cross-reactive antibodies against pandemic (H1N1) 2009 virus, and that 34% of persons born before 1950 had neutralizing titers >80. However, Itoh et al. (*6*) reported that blood donors from Japan who were born after 1920 had almost no appreciable neutralizing antibodies against this virus. Because the hemagglutinin gene of pandemic (H1N1) 2009 virus is similar to that of viruses that circulated in humans during 1918–1957 (*7*), Itoh et al. suggested that pandemic (H1N1) 2009 virus is antigenically divergent from human influenza viruses (H1N1) that circulated during the 1920s–1950s.

Our results are consistent with those of Hancock et al. (*5*), who suggested that human influenza virus (H1N1) circulating in Taiwan after 1920 resembled the 1918 pandemic virus (H1N1) and pandemic (H1N1) 2009 virus and could lead to cross-protection against the current virus. Furthermore, unlike the situation in the United States, there was no program for vaccination against the 1976 swine influenza virus (A/NJ/76) in Taiwan. However, a similar virus was present in Taiwan before 1957. The results of our study also explain why only 7% of patients hospitalized for pandemic (H1N1) 2009 virus in Taiwan were >65 years of age (*8*). Similar epidemiologic observations have been reported in the United States (*3*) and New Zealand (*9*).

Whether elderly persons still have cross-reactivity several decades after exposure to 1918 (H1N1) virus is unknown. The concept of original antigenic sin is a probable explanation. Original antigenic sin has been described in relation to influenza virus, dengue virus, HIV, and several other viruses (*10**–**12*). For persons >65 years of age, the 1918 (H1N1) virus is likely the first influenza virus to which they were exposed, and their antibody response should have increased in subsequent years.

Regression analysis showed that older persons with high HI titers were more likely than younger adults to have higher MN titers. Because the HI assay detects only antibodies against hemagglutinin, the MN assay provides more information about the level of protective antibodies against influenza viruses. As a person ages, production of antibodies against hemagglutinin or other components of influenza virus should protect against potential infections. Although a titer of 40 by HI is accepted as a cutoff value for seroprotection, a consensus for protective titers by MN is lacking (*5**,**13*). We suggest that MN is probably more sensitive than HI for evaluating neutralizing antibodies against pandemic (H1N1) 2009 virus.
